# Mesenchymal Stem Cell-Based Immunomodulation: Properties and Clinical Application

**DOI:** 10.1155/2018/3057624

**Published:** 2018-06-14

**Authors:** Mengyuan Wang, Quan Yuan, Liang Xie

**Affiliations:** State Key Laboratory of Oral Diseases, National Clinical Research Center for Oral Diseases, West China Hospital of Stomatology, Sichuan University, Chengdu 610041, China

## Abstract

Mesenchymal stem cells (MSCs) are multipotent stem cells characterized by self-renewal, production of clonal cell populations, and multilineage differentiation. They exist in nearly all tissues and play a significant role in tissue repair and regeneration. Additionally, MSCs possess wide immunoregulatory properties via interaction with immune cells in both innate and adaptive immune systems, leading to immunosuppression of various effector functions. Numerous bioactive molecules secreted by MSCs, particularly cytokines, growth factors, and chemokines, exert autocrine/paracrine effects that modulate the physiological processes of MSCs. These invaluable virtues of MSCs provide new insight into potential treatments for tissue damage and inflammation. In particular, their extensive immunosuppressive properties are being explored for promising therapeutic application in immune disorders. Recently, clinical trials for MSC-mediated therapies have rapidly developed for immune-related diseases following reports from preclinical studies declaring their therapeutic safety and efficacy. Though immunotherapy of MSCs remains controversial, these clinical trials pave the way for their widespread therapeutic application in immune-based diseases. In this review, we will summarize and update the latest research findings and clinical trials on MSC-based immunomodulation.

## 1. Background

Mesenchymal stem cells (MSCs) are nonhematopoietic stem cells with multipotent properties and self-renewal capability. In addition to bone marrow, MSCs can also be derived from various tissues, including adipose, muscle, umbilical cord blood, peripheral blood, liver, placenta, skin, amniotic fluid, breast milk, synovial membrane, and tooth root [1, 2].

MSCs can act on immune and inflammatory responses following bone marrow-derived MSC-induced T-cell suppression [3]. In addition, MSCs stimulate metabolism, not only through secreting a vast array of chemokines, growth factors, and cytokines but also through production of many secretomes and proteomes. These factors play an important role in immunomodulatory activities, mediating hematopoietic stem cell (HSC) engraftment, and MSC differentiation, as well as regulating angiogenesis and apoptosis. [4]

Because of their remarkable properties for multipotential differentiation and immune mediation, there is potential for using MSCs as a novel therapy for many diseases [5]. Furthermore, MSC-based clinical trials in multiple sclerosis, myocardial infarction, and type 1 diabetes mellitus have been reported [6]. It has also been shown that using soluble factors derived from MSCs improves treatment efficacy for autoimmune disease, which has gained much attention [7]. New insights into the immune-regulatory capacities of MSCs have focused on inflammatory status [8]. The interaction between MSCs and the inflammatory niche furnish vast potential for using MSCs in the treatment of all sorts of diseases, particularly disorders of the immune system [9].

In this review, we will summarize MSC-modulated immunoregulation through description of their constitutive functions, secretion factors, basic functions in regulating immune responses, and clinical value with respect to immunomodulatory treatments.

## 2. Characterization of MSCs

Mesenchymal stem cells have mesodermal lineage differentiation potential and the potential to regulate tissue regeneration [10]. Major characteristics of MSCs include the advantage of multilineage differentiation potential that can generate adipocytes, chondrocytes, and osteocytes due to expression of several pluripotency genes [11–13], thus mediating tissue and organ repair, as well as replacing damaged cells [14].

Currently, MSCs are regarded as a potential new therapy for a variety of human diseases. Recently, studies have focused on regulation of MSC fate with respect to their pluripotency and differentiation to promote regenerative therapeutic development [15, 16]. Increasing numbers of clinical trials are reporting the success of MSC-based immunomodulation based on the measurement of soluble secretors and their interaction with immune cells [17]. Treatment with MSC transplants has attracted much attention based on MSC engraftment studies over the past few years. More importantly, increasing studies have attempted to apply MSCs for the treatment of several autoimmune disorders, such as multiple sclerosis, Crohn's disease, graft versus host disease (GVHD), and systemic lupus erythematosus (SLE) [18].

## 3. MSCs and Immune Modulation

In 2002, it was first shown that MSCs had the ability to modulate immunosuppression by Bartholomew and colleagues, who demonstrated suppression of a mixed lymphocyte response in vitro and prevention of rejection in a baboon skin allograft model in vivo [19]. Since the immune response properties of MSCs were first reported, subsequent studies have shown that MSCs mediate immunosuppression in animal models and human.

Considering the promising preliminary clinical outcomes, the mechanisms of MSC interactions with the immune response as we currently understand them are worth outlining. MSCs have the ability to interact with many kinds of immune cells, including B cells, T cells, dendritic cells (DCs), natural killer (NK) cells, neutrophil, and macrophages [20]. Mechanisms of interaction were shown to rely on cell–cell contact working in collaboration with secretion of soluble immune factors to induce MSC-regulated immunosuppression [21]. These specific modulators, including a multitude of immune-modulatory factors, cytokines, and growth factors, modulate inflammatory responses and balance immune profiles [22]. Namely, soluble immune secretomes, such as prostaglandin E2 (PGE-2), indoleamine 2,3-dioxygenase (IDO), or nitric oxide (NO), respond to immune cells to activate immunoregulation by MSCs [23].

Adhesion molecules, intracellular secretomes, and the main histocompatibility complex (MHC) antigens are all required to induce immune suppression. Particularly T cells, as well as the Fas ligand/Fas receptor interaction (FasL/FasR), play a vital role in T-cell reaction function [24]. Extracellular vesicles produced by MSCs accelerate generation of M2 macrophages and regulatory T cells, while at the same time suppressing maturation of monocytes and proliferation of T cells and B cells [17, 25].

MSCs also have the ability to regulate inflammatory progress and repair damaged cells and tissues by adhering to inflammatory sites [26]. MSC integration with inflammatory actions can both fortify and restrain the immune response and is dependent on the function of immune suppressants, the kinds of inflammatory secretomes, and the general condition of the immune system [27]. Interestingly, MSCs only modulate immunosuppression when they are first stimulated by inflammatory cytokines, such as tumor necrosis factor (TNF) and interleukin- (IL-) 1 [28]. MSCs not only respond to inflammatory cytokines but also produce immune-regulatory secretors that mediate the process of inflammation. For example, a large number of indoleamine 2,3-dioxygenase (IDO) in humans, nitric oxide (NO) in mice, and chemokines produced by MSCs play a key part in MSC-mediated immunomodulation [29]. Furthermore, MSC secretomes, including growth factors hepatocyte growth factor (HGF) or tumor-specific glycoprotein (TSG6), have been effectively utilized to treat immune diseases [30]. MSCs themselves have also been used to successfully treat patients with severe immune disorders, including Crohn's disease and SLE [31].

### 3.1. Immune Cells Interact with MSCs in Immunomodulation

Both in vivo and in vitro studies have shown that MSCs present their multipotency as a mediator of immunomodulation. MSCs exert significant effects on immunosuppression by refraining immune cells in both the innate and adaptive immune systems (Table 1).

#### 3.1.1. Innate Immunity

The innate immune system plays a pivotal role not only in the adaptive immune reaction but also in the elimination of pathogens targeted by an adaptive immune response [32]. DCs, NK cells, and macrophages constitute the innate immune system, and their interaction with MSCs promotes regenerative processes and inhibits inflammatory responses [33].

*(1) Myeloid Dendritic Cells (DCs).* Myeloid dendritic cells (DCs) maintain and modulate immune responses through acceleration of antigen-specific T-cell processes, as well as activation of cells in the innate immune reaction following DC maturation [34, 35]. Recent studies demonstrated that MSCs have immunosuppressive functions on DCs in the form of restraining DC differentiation from monocytes and decreasing the cell-surface expression of CD1-*α*, CD40, CD80, CD83, CD86, and MHCII [36]. After incubation with MSCs, DCs would lose their capability to motivate lymphocytes by downregulating interferon-*γ* (IFN-*γ*) and TNF-*α* expression as well as accelerating IL-10 release [37]. The MSC-DC interaction is mediated by the Notch pathway relied on IFN-*γ*-secretase [38]. The molecular mechanisms of MSCs restraining DC maturation seem to be regulated by PGE-2 [39]. In addition, MSCs have the ability to damage the migration of DCs by suppressing molecules tied to DCs and presenting antigens for activating T cells [40, 41]. MSCs can also depress the proinflammatory capacity of DCs by inhibiting the formation of TNF [4]. Importantly, the inhibitory effects of MSCs play a significant role in relieving some immune disorders, such as allograft rejection [38], type 1 diabetes, and acute GVHD [42–44].

*(2) Natural Killer (NK) Cells.* Natural killer (NK) cells produce proinflammatory cytokines and have cytolytic activity [45].

MSCs inhibit the effects of NK cells with immunosuppressive secretors, such as PGE2, TGF-*β*, and sHLA-G, leading to induction of cytotoxic effects against virus-infected cells and reduction of IFN-*γ* secretion [46]. This inhibitory action is completed by suppressing the activating NK-cell receptor expression, which is mediated by IDO and PGE-2 [47]. In addition to these findings, direct cell–cell contact also plays a distinct role in suppressing NK cells, which is related to expression of Toll-like receptor- (TLR-) 4 on MSCs [48]. MSCs promote cytotoxic movement by suppressing the secretion of NKp30 and NKG2D, which are the surface receptors related to NK-cell activation [49]. However, the potent suppressive actions of MSCs were only apparent at high MSC-to-NK ratios [46]. Furthermore, it has been demonstrated that activated NK cells have the ability to dissolve MSCs when there are enough activating receptors on NK cells [50]. Together, these discoveries indicate that interaction between MSCs and NK cells relies on the ratios of both cells, as well as their microenvironment [3].

*(3) Macrophages.* It is well known that macrophages are important cells in the innate immune system with significant plasticity [51]. Based on the specific microenvironment of MSCs, macrophages may be polarized into classically activated M1 macrophages or alternatively activated M2 macrophages [52]. Generally, M1 macrophages possess prominent antimicrobial properties by releasing a variety of chemokines and inflammatory cytokines, whereas M2 macrophages are able to alleviate inflammation and expedite tissue repair via secretion of IL-10 and trophic factors [53]. In addition, the coculture of macrophages with MSCs induces production of M2 macrophages, which upregulates the phagocytic activity and secretion of IL-10, and downregulates levels of inflammatory cytokines, such as IFN*γ*, TNF-*α*, IL-1*β*, and IL-12 [54, 55]. Recent studies reported that MSCs facilitated monocyte chemotactic protein-1 (MCP1) secretion by responding to TLR4 ligation, then inducing monocyte emigration [56]. In a model of zymosan-induced peritonitis injury, human MSCs activate peritoneal macrophages by secreting TNF-stimulated gene 6 (TSG6), which regulates TLR2 nuclear factor-*κ*B (NF-*κ*B) signaling [57]. Additionally, MSCs have been shown to ameliorate immune disorders and accelerate tissue regeneration by increasing the concentration of macrophages at locations of injury [58, 59].

#### 3.1.2. Adaptive Immunity

The adaptive immune system has its own distinct properties, specifically antigen-specific immune response and immunological memory. The system consists of CD4^+^ T helper and CD8^+^ cytotoxic T lymphocytes that transmit a suitable antigen-specific immune response after antigen-presenting cells (APCs) undergo antigen processing and presentation [32].

*(1) T Cells.* T cells are widely distributed in both animal and human tissues and, once activated, can differentiate into T helper (Th) 1, regulatory T cell (Treg) subpopulation, Th2, Th9, or Th17, according to the intensity of stimulation and the cytokine microenvironment [60, 61]. It has been demonstrated that MSCs interact tightly with T cells [62]. More importantly, as a key mediator of the adaptive immune system, T cells modulate various autoimmune diseases and protect organisms from infections and malignancies [63].

On the other hand, MSCs secrete a great quantity of immunosuppressive factors, chemokines, and adhesion molecules, which are responsible for effective T-cell suppression, involved in T-cell proliferation and apoptosis, as well as differentiation [26, 64]. For example, MSCs are capable of repressing T-cell proliferation through cellular or nonspecific mitogenic stimuli [65] and promoting apoptosis of activated T cells via the Fas/Fas ligand pathway [66]. MSCs constitutively secrete coinhibitory molecule B7-H4 and HLA-G, which present an immunosuppressive action on T cell and influence their proliferation as well as T cell-mediated cytotoxicity [67]. However, the immunosuppressive capacity of MSCs is not activated at all times and relies on the strength and type of the inflammatory stimulation [68]. MSCs no longer restrain T-cell proliferation in the presence of pathogen-associated molecules and TLRs such as TLR3 and TLR4 which damage Notch signaling, thereby recovering effective T cell to respond to pathogens [69]. In addition, regulatory T cells, as a specialized subset of T cells, restrain effects of the immune system, leading to relieving their own antigens and sustaining homeostasis [70].

*(2) B Cells.* B cells are the second major cell genre related to adaptive immune responses. These cells resist and hunt down outside pathogens through the production of specific antibodies [71, 72]. Both murine and human MSCs have the ability to inhibit B-cell proliferation and activation in vitro [73]. Additionally, MSCs also suppress differentiation of B cells, as well as expression of chemokine receptors owing to cell–cell contact and secretion of soluble molecules [74]. Metalloproteinase-processed CC-chemokine ligand 2(CCL2) released by MSCs suppress signal transducer and activator of transcription 3 (STAT3) activity, resulting in downregulating Paired box 5 (PAX5), thereby inhibiting immunoglobulin synthesis [75]. Several other signaling pathways, such as p38, extracellular response kinase 1/2, B lymphocyte-induced maturation protein 1 (Blimp1), and Akt signaling also modulate B-cell activation [76]. However, inadequate inflammatory signal-activated MSCs from patients with SLE may support proliferation and differentiation of antibody-releasing B cells [77]. Taken together, MSCs suppress antibody production by B cells, and this effect is dependent upon the strength of the inflammatory stimulation, as well as the ratio of MSCs to B cells [78, 79].

### 3.2. Soluble Factors Secreted by MSCs in Immunomodulation

MSCs could interact with immune cells in both the innate and adaptive immune systems by secreting multiple soluble immune factors to induce MSC-regulated immunosuppression [80]. During an immune response, a number of soluble factors are released by MSCs, such as cytokines, growth factors, hormones, and chemokines, which act on immune cells and exert their functions by repairing damaged cells or suppressing immunology activity [81, 82] (Table 2). The inflammatory response is pivotal for MSCs to exert effects on immunomodulation, owing to an inflammatory cytokine-licensing process by MSCs. Consequently, the immunoregulatory activities of MSCs require inflammatory cytokines secreted by antigen-presenting cells and T cells, which include interferon- (IFN-) *γ*, IL-1*α*, IL-1*β*, and TNF-*α* [83]. These inflammatory cytokines can activate MSCs to secrete immunosuppressive factors consisting of IDO, TSG6, NO, IL-10, CCL2, galectins, PGE2, and TGF-*β* and then modulating tissue homeostasis [13, 25].

#### 3.2.1. Indoleamine 2,3-Dioxygenase (IDO)

Recently, it has been reported that indoleamine 2,3-dioxygenase (IDO) mediates immunomodulation by suppressing various immune cells, including T cells and NK cells [78, 84]. IDO can restrain the proliferation and effect of immune cells by transforming tryptophan into its metabolite kynurenine [85]. Furthermore, IDO secreted by MSCs has the ability to suppress allogeneic T-cell reactivity and promotes kidney allograft tolerance [86]. In addition, IDO has been proposed to be one of the representative immunosuppressive molecules for human MSCs [78, 87].

#### 3.2.2. TNF-Stimulated Gene 6 (TSG6)

TNF-stimulated gene 6 (TSG6) is a multifunctional protein with anti-inflammatory effects [88]. Proinflammatory mediators, such as TNF-a and IL-1, may stimulate the secretion of TSG6 [89]. It has been reported that microembolization induces TSG6 to interact with damaged lung in a mouse model of myocardial infarction. Thus, TSG6 plays a significant role in reducing inflammation and infarct size, as well as enhancing cardiac function [7].

#### 3.2.3. NO

In the presence of proinflammatory cytokines, MSCs facilitate high expression of inducible NO synthase (iNOS), which stimulates the secretion of NO, giving rise to inhibition of T-cell proliferation [90]. Both in vivo and in vitro studies showed that murine MSCs lacking iNOS exhibited diminished inhibition capability [26]. Intriguingly, high concentrations of NO may suppress immune modulation and lead to immune cell apoptosis via inhibition of signal transducer and activator of transcription 5 (STAT5) phosphorylation and signal transducer in T cells [90, 91]. However, NO is an extremely unstable oxidative molecule, and both adhesion molecules and chemokines can assist it in exerting immunosuppressive action [77, 92].

#### 3.2.4. IL-10

IL-10 was reported to play a crucial part in MSC-regulated immunosuppression [4]. Antigen-presenting cells, including monocytes and dendritic cells, could work with MSCs to induce secretion of IL-10 [93]. In addition, Macrophages can deliver large quantities of IL-10 by stimulation of E prostanoid receptors, thereby protecting tissues against migration of neutrophils [94].

#### 3.2.5. CC-Chemokine Ligand 2 (CCL2)

CC-chemokine ligand 2 (CCL2), as a metalloproteinase-processed chemokine, antagonizes the function of CC-chemokine receptor 2 (CCR2), which is the cognate receptor of CCL2 [95]. Binding of CCL2 to CCR2 has been shown to mediate immunosuppression of MSCs by inhibiting activation and migration effects on TH17 cells in experimental autoimmune encephalomyelitis (EAE) [96]. Furthermore, CCL2 secreted by mouse MSCs accelerates monocyte migration from the bone marrow into the blood stream, verifying the notion that MSC interaction with innate immune responses affects the immune system [96].

#### 3.2.6. Prostaglandin E2 (PGE2)

Prostaglandin E2 (PGE2) is another immunosuppressive factor secreted by inflammatory stimulus-induced MSCs. PGE2 regulates immunosuppression of MSCs in T cells, DCs, NK cells, and macrophages [83, 84]. In vitro, PGE2 produced by mouse MSCs restrain several cell functions, such as TNF generation and migration [97]. Additionally, in an experimental mouse model of sepsis, IL-10-dependent PGE2 has been described to play a significant role in treating effectively mice with MSCs [83]. More significantly, PGE2 collaboration with IDO exerts immunosuppressive actions in human MSCs, such as inhibiting T-cell proliferation, as well as NK cell cytolytic activity [84].

It seems that all these molecules exert their functions in reliance on the inflammatory microenvironment. Therefore, future research should focus on mediator mechanisms that regulate the immunosuppressive characteristics of MSCs, as well as their local microenvironments, which will provide a broad perspective for therapeutic application of MSCs [13].

## 4. MSC Clinical Applications in Immune-Mediated Disease

Since MSCs derived from bone marrow were first suggested for use in regenerative medicine owing to their stem cell-like qualities, there have been many major studies on applying the multipotential capacity of MSCs in promoting transplanted HSC engraftment and facilitating damaged tissue repair [98]. The immunosuppressive capacities of MSCs have provided new insight for the treatment of immune-mediated diseases. In addition to restraining immunocompetent cells by suppressing their response to antigen and sustaining them in a silent state, MSCs also promote peripheral tolerance [3]. In addition, MSCs could induce T-cell tolerance and damage pathogenic T- and B-cell response.

MSCs bring new vitality to the study of various immune disorder-related diseases and tissue regeneration through their immune-regulatory properties. Some studies have verified that MSCs induce tissue regeneration in the liver [99], kidney [100], and heart [101]. These damaged tissues may be directly replaced by MSCs with multipotent differentiation abilities. Additionally, MSCs can exert immune-regulatory capabilities to treat immunological disorders by decreasing inflammation and promoting tissue repair. For instance, the inhibitory function of MSCs contributes to relieving several immune disorders, including peritonitis, endotoxemia, type 1 diabetes, type 2 diabetes, acute GVHD, ischemia–reperfusion injury, acute liver injury, arthritis, allograft rejection, and atherosclerosis [102].

In fact, a variety of immune disorder diseases, including multiple sclerosis, Crohn's disease, GVHD, SLEs, and type 1 diabetes, have entered clinical trials for treatment with MSCs [80]. Moreover, prochymal and cupistem products based on the immunomodulatory capabilities of MSCs have been widely applied for disease therapy in a number of disorders [103].

More importantly, recent preclinical and human studies support the hypothesis that MSCs derived from allogeneic donors could be utilized in clinical therapy [104]. Furthermore, in many subacute conditions, as in autoimmune diseases, there is enough time to obtain and culture autologous MSCs in vitro, whereas allogeneic MSCs may be the only option for major acute conditions [105].

There have been over 700 MSC-based clinical trials registered on the National Institutes of Health (NIH) Clinical Trial Databank (https://clinicaltrials.gov/) around the world as of the end of October 2017. Surprisingly, although the immunomodulatory capacities of MSCs have been only recently confirmed, MSC-based therapies have quickly risen in prominence among immunology disease treatments in the past few years. There are 105 clinical trials related to the immunomodulatory effects of MSC and 44 clinical trials linked to graft enhancement, utilizing the immunosuppressive functions of MSCs (Table 3).

**Figure 1:**
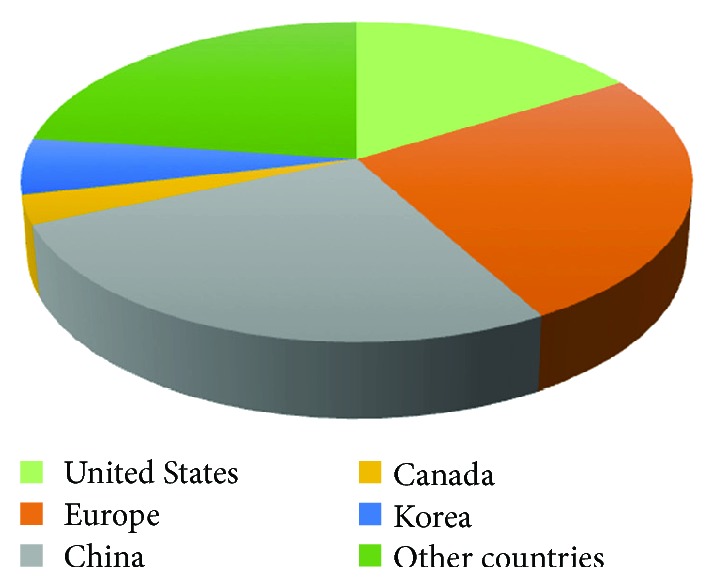
The clinical trial distribution of MSC-based immunomodulation in the world. Up to now, most of the clinical trials using MSCs for treating inflammatory or autoimmune diseases have been conducted in the China, US, and Europe. All values have been extracted from https://clinicaltrials.gov/.

To date, most of the MSC-based clinical trials related to immunomodulation have been administered in China and Europe, as well as the United States (Figure 1). The major clinical indications within the clinical trial database consist of multiple sclerosis/atherosclerosis (*n* = 31), Type 1 diabetes (*n* = 18), Crohn's disease (*n* = 22), systemic lupus erythematosus (*n* = 11), and GvHD (*n* = 41) (Table 1).

### 4.1. Graft versus Host Disease (GVHD)

The first successful MSC therapy case comprised infusion of MSCs obtained from bone marrow into an IV for an acute GVHD-grade patient with cyclosporine- and steroid-resistant features [106]. HSC remains the most successful treatment among all stem cell therapies [102]. However, in spite of the immunosuppressive effects of allogeneic HSC transplantation, immune rejection still causes 30–40% morbidity and mortality in GVHD therapeutic applications [107]. GVHD pathogenesis includes an alloresponse to donor lymphocytes, which leads to damage in multiple organs, especially liver, skin, and gastrointestinal tract [108]. Importantly, the treatment for GVHD using MSCs developed more quickly than for other immune-based diseases [106]. Published reports from completed clinical trials included the conclusion that MSCs to treat GVHD decreased the 2-year mortality rate [109]. These hopeful results provide valuable resources for larger-scale clinical trials. Recently, the mesoblast has received fast-track designation for MSC-100-IV for treatment of steroid-refractory acute GVHD in children, so far this allogeneic mesenchymal stem cell (MSC) product has completed enrollment and topline results are expected in 2018. MSCs have their own advantages in the treatment of GVHD; however, both strict standard MSC processing and precise tailoring for patients are necessary to reach coherent and repeatable results.

### 4.2. Crohn's Disease

In the early 1990s, Crohn's disease patients were reported to experience relief from their inflammatory bowel disease following infusion of HSCs [110]. Subsequently, the development of HSC transplantation has been widely applied in Crohn's disease therapy [111]. However, serious adverse effects also accompanied HSC transplantation treatment. More importantly, MSCs have the privilege of exerting immune-mediated actions without a requirement for host-recipient matching, attributed to low expression of HLA class I antigen and HLA class II antigen on MSC surfaces [57, 112]. Early phase studies have demonstrated that allogeneic MSCs exert effects on luminal disease, while either allogeneic or autologous MSCs exhibit efficacy on fistula disease [113]. A phase 3 study is now evaluating the efficacy of allogenic adipose-derived MSC in 212 patients to treat refractory, perianal fistulizing Crohn's disease. The primary end point showed that remarkably more patients treated with MSC had achieved complete closure of fistula at 24 weeks [114]. In addition, TiGenix following phase 3 has successfully achieved European Medicines Agency (EMA) approval for their allogeneic MSCs being developed to treat complex patients with Crohn's disease. MSCs have extensive application prospects for Crohn's disease treatment, but future work should focus on defining accurate phenotypic and functional features of conducted cells.

### 4.3. Multiple Sclerosis (MS)

Multiple sclerosis (MS) is a CD4 T cell-modulated autoimmune disease dependent on myelin-based protein (MBP), a protein discovered uniquely in myelin sheaths [115]. Currently, MS is one of the most prevalent autoimmune diseases mediated by CD4 T cells in the central nervous system (CNS). Astonishingly, there are 2.3 million people influenced by this disease that has no cure [116]. It has been demonstrated that MSCs exert productive therapeutic effects in an EAE mouse model, one of the best MS models [117]. Furthermore, both demyelinating regions and lymphoid organs could be used to test the presence of either human or mouse MSCs by intravenous infusion in the EAE mouse model [117, 118]. Adipose-derived MSCs decrease the symptoms of EAE and inflammation of spinal cord and brain according to MSCs targeting in CNS and lymphoid organs [119]. It has also been demonstrated that intravenous administration of human MSCs could prolong EAE mice life and ameliorate the disease by the mechanisms of immunomodulation [120]. Various preclinical investigations have reported MSC therapeutic efficacy in animal models for MS. Up to now, there have been 31 clinical trials of MS registered on the NIH Clinical Trial Database (Table 1), which are researching the MSC effectiveness and adverse effects for treatment MS patients all over the world, and a clinical trial has reported the validity of intravenous administration of MSCs from autologous bone marrow for patients with MS [121]. However, there are no significant positive achievements reached until now, though conducting many phase I/II researches and further investigations are needed before they can be widely applied to clinical treatment.

### 4.4. Type 1 Diabetes

Diabetes mellitus is a common metabolic disease, with nearly 171 million adult patients around the world [122]. Type 1A diabetes mellitus is characterized by T cell-mediated autoimmune disorder, giving rise to the destruction of pancreatic *β* cells, as well as decreasing insulin secretion owing to existing anti-islet cell antibodies [123]. Currently, insulin injections and blood glucose control is still the standard therapeutic methods for type 1 diabetes [124]. However, the urgent need for a new option to treat type 1 diabetes is necessary due to the adverse effects accompanied by insulin treatment and the difficulties in maintaining metabolic balance.

In fact, compared with other stem cells, MSCs have more advantages for therapeutic application in type 1 diabetes owing to their immunosuppressive ability and multilineage differentiation [125]. The preliminary results indicated that MSCs derived from the umbilical cord blood reverse the autoimmune that could regenerate islet beta cells and reinforce glycemic control of type 1 diabetes [126]. Human bone marrow-derived MSCs took effects on immune tolerance in a mouse model transplanted with human islets [127]. Furthermore, Urban's study has suggested that MSCs may be a new way to treat insulin-dependent diabetes, and these encouraging results in vivo and in vitro provide enthusiasm for MSC-based theoretical use in clinical trials [128]. However, some cautions should be focused on MSC transformation and tumorigenicity with passages due to the gene mutation.

### 4.5. Systemic Lupus Erythematosus (SLE)

Systemic lupus erythematosus (SLE) is also a chronic autoimmune disease characterized by a multiorgan inflammatory response [129]. Both the proinflammatory cytokine stimulation and autoantibody complex production in SLE bring about activating immune cells in both innate and adaptive immune systems. Over the past 50 years, there is only one drug permitted by the USA Food and Drug Administration (FDA) for the treatment of SLE disease that remains controversial [130]. Consequently, new treatments targeting immune intervention would represent significant advantages for SLE patients. Recently reports have suggested that extracellular vesicle (EV) secreted from MSCs could be used as a cell-free therapy. The preclinical results showed MSC-derived EVs inhibit inflammatory responses and suppress autoimmune disease pathogenesis [131]. Most promising animal studies' data and clinical trials about SLE treatment promoted MSC application in the therapy of SLE patients [131]. The Nanjing Drum Tower Hospital in China has used MSCs to treat over 300 refractory SLE patients. Their results showed 32.5% patients reached a significant clinical efficacy with a well-tolerated safety and a dramatic decline in disease activity scores. And the group published that SLE patients transplanted with MSCs achieved remarkable effect and long-term safety at a 4-year review [113]. However, some clinical trials reported that MSCs convey tumorigenic potential, and the immunosuppressive efficacy could be influenced by donor alteration as well as ex vivo amplification. [102]

## 5. Controversy over Mesenchymal Stem Cell Therapy

Currently, MSC treatment has already entered clinical trials for the treatment of organ transplantations, tissue regeneration, and autoimmune diseases [132]. Even though the FDA appointed that MSC transplantation was safe, recent researches stated that potential long-term risks involved in MSC treatment may have not appeared in the short term [133]. Many possible complications of MSC therapy are associated with immunosuppressive properties of MSCs on account of reduction of immunosurveillance to host and foreign pathogens or virus [134]. Furthermore, autologous MSCs may, in theory, induce tumors by changing the action of cancer cells and accelerating tumor cell growth. In addition, allogeneic MSCs derived from donors may accelerate infectious risk [135]. Notable questions need to be concentrated about MSCs on the durability of response and long-term safety. For example, less is known about the route administration of MSC home to the site of inflammation and survival in tissues. Furthermore, delivery dose, safety control, as well as optimized standards for MSC derivation and amplification are also needed to be explored for the better development of MSC-based clinical trials [136].

## 6. Conclusion

MSCs are multipotent progenitor cells with multilineage differentiation potential and immune-modulatory properties. MSCs have the ability to interact with immune cells both in innate and adaptive immune systems. In addition, MSC-mediated immunosuppression is dependent on the combined reaction of chemokines, inflammatory cytokines, and effector factors secreted by MSCs, as well as the microenvironment and strength of the inflammatory stimulus [137]. In the past few years, emerging data have demonstrated that MSCs exert immunoregulatory effects that provide a promising tool for the therapy of tissue repair, inflammatory diseases, and immune disorders [3]. MSCs showed unique advantages and achieved significant improvements in the therapy of immune diseases. Nevertheless, MSC-based treatment does not always provide advantages according to tightly interacting with the microenvironmental milieu. The risks of MSC therapy conclude potential complications of immunosuppression, ectopical differentiation, and promotion of tumor growth [138]. In consequence, many issues need to be settled before clinical application of MSCs [23]. Even though these immunomodulatory characteristics are not entirely elucidated, the immunosuppressive potential of MSCs makes them a promising therapy for various autoimmune diseases [20]. We believe further investigation of MSC-based molecular mechanisms as well as additional clinical trials will enhance our understanding of MSC immunomodulation and aid in the development of clinical applications utilizing MSC treatment [136].

## Figures and Tables

**Table 1:** The function of MSCs in mediating immune cells both in innate and adaptive immune systems.

Immune cell type	MSC functions
Innate immune systems	
DCs	Inhibiting DC migration, activation, differentiation, maturation, and endocytosis
NK cell	Inhibiting NK cell migration, proliferation, differentiation, maturation, and activation
Macrophage	Activating M2 macrophage polarization in general; activating M1 macrophage polarization in specific microenvironment

Adaptive immune systems	
T cell	Inhibiting T-cell survival proliferation, differentiation, maturation, and activation, while accelerating T-cell recruitment
B cell	Inhibiting B-cell proliferation, differentiation, maturation, chemotaxis, and activation

**Table 2:** Biological function of soluble factor secreted by MSCs.

Soluble factors	Biological function
IDO	Suppressing proliferation and effect of immune cells
TSG6	Anti-inflammatory effect
NO	Suppressing proliferation and modulation of T cell, promoting apoptosis of immune cells
IL-10	Suppressing apoptosis of immune cells
CCL2	Suppressing activation and migration of TH17 cells, promoting migration of monocyte
PGE2	Suppressing generation and migration of TNF, proliferation of T cell, and cytolytic activity of NK cell

**Table 3:** Clinical trials using mesenchymal stem cells (registered as of October 26, 2017).

Indication	Number of studies
*Immunomodulation*	*105*
Multiple sclerosis/atherosclerosis	31
Type 1 diabetes	18
Crohn's disease	22
Systemic lupus (erythematosus/colitis)	11
Rheumatoid arthritis/Sjögren's syndrome	7
Buerger's disease/sickle cell disease	3
HIV	2
Limbus corneae insufficiency syndrome	1
Periodontitis	5
Progressive hemifacial atrophy	2
Retinitis pigmentosa	3
*Graft enhancement*	*44*
GvHD	41
Hematopoietic malignancies	3

All values have been extracted from https://clinicaltrials.gov/.

## Abbreviations

MSCs: Mesenchymal stem cells
HSC: Hematopoietic stem cell
GVHD: Graft versus host disease
SLE: Systemic lupus erythematosus
TNF: Tumor necrosis factor
IL: Interleukin
IDO: Indoleamine2,3-dioxigenase
NO: Nitric oxide
HGF: Hepatocyte growth factor
TSG6: TNF-stimulated gene 6
DCs: Dendritic cells
NK cells: Natural killer cells
PGE-2: Prostaglandin E2
MHC: Main histocompatibility complex
FasL/FasR: Fas ligand/Fas receptor interaction
IFN-*γ*: Interferon-*γ*
EGF: Epidermal growth factor
MCP1: Monocyte chemotactic protein-1
NF-*κ*B: Nuclear factor-*κ*B
PAX5: Paired box 5
Blimp1: B lymphocyte-induced maturation protein 1
FGF: Fibroblast growth factor
PDGF: Platelet-derived growth factor
VEGF: Vascular endothelial growth factor
IGF-1: Insulin-like growth Factor-1
SDF-1: Stromal cell-derived factor 1
ICAM: Intercellular cell adhesion molecule
HLA: Human leukocyte antigen
VCAM: Vascular cell adhesion molecule
CCL2: CC-chemokine ligand 2
iNOS: Inducible NO synthase
STAT5: Signal transducer and activator of transcription 5
CCR2: CC-chemokine receptor 2
EAE: Experimental autoimmune encephalomyelitis
TLR: Toll-like receptor
APCs: Antigen-presenting cells
Th: T helper
Treg: Regulatory T cell
MS: Multiple sclerosis
MBP: Myelin-based protein
CNS: Central nervous system
FDA: Food and Drug Administration
NIH: National Institutes of Health
EV: Extracellular vesicle.
